# Association of sleep time with all-cause and cardiovascular mortality among individuals with advanced cardiovascular-kidney-metabolic syndrome: A NHANES-based prospective cohort study

**DOI:** 10.1097/MD.0000000000051259

**Published:** 2026-10-23

**Authors:** Yangyang Xiong, Yue Yin, Jing Wang, Ke Lan, Jiahua Liang, Wencong Han

**Affiliations:** a School of Medicine/Clinical Medicine, Guangzhou University of Chinese Medicine, Guangzhou, Guangdong, China; b Meizhou Hospital, Guangzhou University of Chinese Medicine, Meizhou, Guangdong, China; c School of Medicine, Hubei University of Chinese Medicine, Wuhan, Hubei, China; d Department of Medicine/Health, Lingnan Vocational and Technical College, Qingyuan, Guangdong, China; e Fourth Clinical Medical College, Guangzhou University of Chinese Medicine, Shenzhen, Guangdong, China.

**Keywords:** all-cause mortality, cardiovascular mortality, cardiovascular-kidney-metabolic syndrome, NHANES, sleep time

## Abstract

To delve into the relationship of sleep time with the onset of all-cause and mortality from cardiovascular disease (CVD) among individuals suffering from advanced Cardiovascular-Kidney-Metabolic (CKM) syndrome. This prospective cohort study collected information from the National Health and Nutrition Examination Survey (NHANES) 2005 to 2018 that recruited 4266 adult participants with advanced (Stage 3–4) CKM syndrome. Sleep time was measured as the self-reported sleep hours and classified as <7 hours, 7 to 8 hours (reference group), and >8 hours per night. Mortality outcomes including all-cause and cardiovascular death were ascertained by linking records to the National Death Index (NDI) up to December 31, 2019. Kaplan–Meier survival curves analyzed mortality by sleep time group. In the study, the researchers investigated the relationship between sleep time and mortality utilizing multivariable Cox proportional hazards models. Besides, restricted cubic spline analysis was performed to check for possible nonlinear relationship between sleep time and all-cause/CVD death. A total of 1294 all-cause death cases and 465 due to CVD within a median 7.6 years of follow-up. Within the completely adapted model by Cox, comparison with a reference group (7–8 hours), long sleep time (>8 hours) was linked to a significantly increased CVD death risk (Hazard Ratio [HR]: 1.53; 95% confidence Interval: 1.14–2.07; *P = .005*) and showed a borderline remarkable connection with all-cause death (HR: 1.21; 95% confidence Interval: 1.00–1.46; *P = .05*). Short sleep time (<7 hours) did not significantly correlate with either death outcome. The analysis revealed U-shaped association of sleep time with overall mortality (*P for nonlinearity = .0017*). In the end, sleep time in patients with advanced CKM syndrome exhibited a U-shaped or J-shaped connection with death rates. Sleeping too much (>8 hours) has been deemed a pronounced risk indicator for both CVD and overall mortality. According to these results, sleep time ought to be seen as an essential health marker in managing advanced CKM syndrome clinically and long-sleepers require additional clinical assessment.

## 1. Introduction

Sleep represents an essential physiological activity that is important for public health as well as people’s overall well-being. Serving as an innate adaptation to the day-night cycle, this physiological process is modulated by sophisticated neural networks and conforms to circadian rhythmic patterns.^[1]^ To have good sleep for adults, the National Sleep Foundation has suggested a sleep time of 7 to 8 hours.^[2]^ Nevertheless, in the modern world, where things are accumulating quickly and stress is becoming the new normal, many people are now always short of sleep or sleep too much due to going on night shifts, bad lifestyle habits, more use of electronic gadgets, etc^[3,4]^ The American Heart Association (AHA) has defined Cardiovascular-Kidney-Metabolic (CKM) syndrome in 2023 to be a multifactorial systemic condition. It arises from pathological cross-talk between obesity and related metabolic risk states, including chronic kidney disease, diabetes, as well as cardiovascular lesions.^[5,6]^ According to global health data for 2021, stroke and ischemic heart disease are the first disease burdens for patients with CKM syndrome.^[7]^ The poor health status associated with CKM syndrome is a primary determinant of overall mortality worsening the public health and socioeconomic burden globally.

A rising number of epidemiological studies as well as prospective cohort studies at present have demonstrated that sleep time has a major influence on cardiovascular and renal as well as metabolic health. There exists a nonlinear connection between sleep time and death with various causes.^[8–23]^ Moreover, shortened or prolonged sleep times have been found to correlate with CKD, metabolic syndrome, as well as metabolic abnormalities like central obesity, hyperglycemia, and dyslipidemia.^[24–34]^ Some studies have evaluated the relationship between the CKM syndrome (and its development) and sleep quality. Researchers suggest that sleep health may offer some protection against the progression of CKM syndrome.^[35]^ There are currently no large-scale prospective cohort studies evaluating the connection between sleep time and death rates (both all-cause and cardiovascular) in individuals suffering from advanced CKM syndrome. Given the high-risk characteristic of this particular population and lack of evidence available, further clarifying the connection between sleep time and risk of death in advanced CKM syndrome holds particular significance for directing clinical management and developing individualized intervention strategies.

We accessed the information from National Health and Nutrition Examination Survey (NHANES) regarding NDI documents to study the connection between sleep time and overall as well as cardiovascular death among advanced CKM syndrome Patients. We hypothesized that duration of sleep that was either excessively short or excessively long might contribute to more frequent all-cause and cardiovascular fatal outcomes among individuals suffering from advanced CKM syndrome. In addition, the present study determined relevant factors that influence sleep time in this population. Through a systematic assessment of this association, we provided a theory for rational management of sleep time, mortality risk assessment, and optimization of preventive measures for CKM syndrome patients. The research results could provide more efficient health management methods for adult patients suffering from advanced CKM syndrome. Furthermore, they could serve as references for dealing with later sleep-related matters.

## 2. Methods

### 2.1. Research population and design

NHANES was hereby used as the data source (https://www.cdc.gov/nchs/nhanes/index.htm).^[36]^ Operated by the National Center for Health Statistics (NCHS) under the US Centers for Disease Control and Prevention, NHANES represents a population-based cross-sectional investigation. This survey adopts stratified multi-stage cluster random sampling to evaluate physical health status and nutritional conditions among American residents of all age groups. The surveys cover everything from demographics to socioeconomic factors, dietary and health issues and much more. The NCHS Institutional Review Board supported the research protocol, which is consistent with the Declaration of Helsinki. These participants all offered their informed consent. The NHANES cycles (2005–2018) first recognized 6264 participants aged over 20 years and with Stage 3 to 4 CKM syndrome for analysis. The patients were removed in sequence according to the following factors: being pregnant at baseline (n = 6); having missing data for sleep time (n = 44); missing follow-up data (n = 11); missing other relevant covariates (total n = 1937); marital status (n = 2); poverty-to-income ratio (PIR, n = 603); education level (n = 6); alcohol use (n = 525); dietary data (n = 190); body mass index (BMI, n = 351); estimated glomerular filtration rate (eGFR, n = 179); total cholesterol (TC, n = 6); (systolic blood pressure [SBP], n = 73); history of malignancy (n = 2). In the end, a total of 4266 participants were finally enrolled for subsequent analysis. A comparison of baseline characteristics between included and excluded participants is provided in Supplementary Table S1, Supplemental Digital Content 1. The flowchart of participant recruitment, including detailed inclusion and exclusion criteria, is presented in Figure 1.

**Figure 1.**
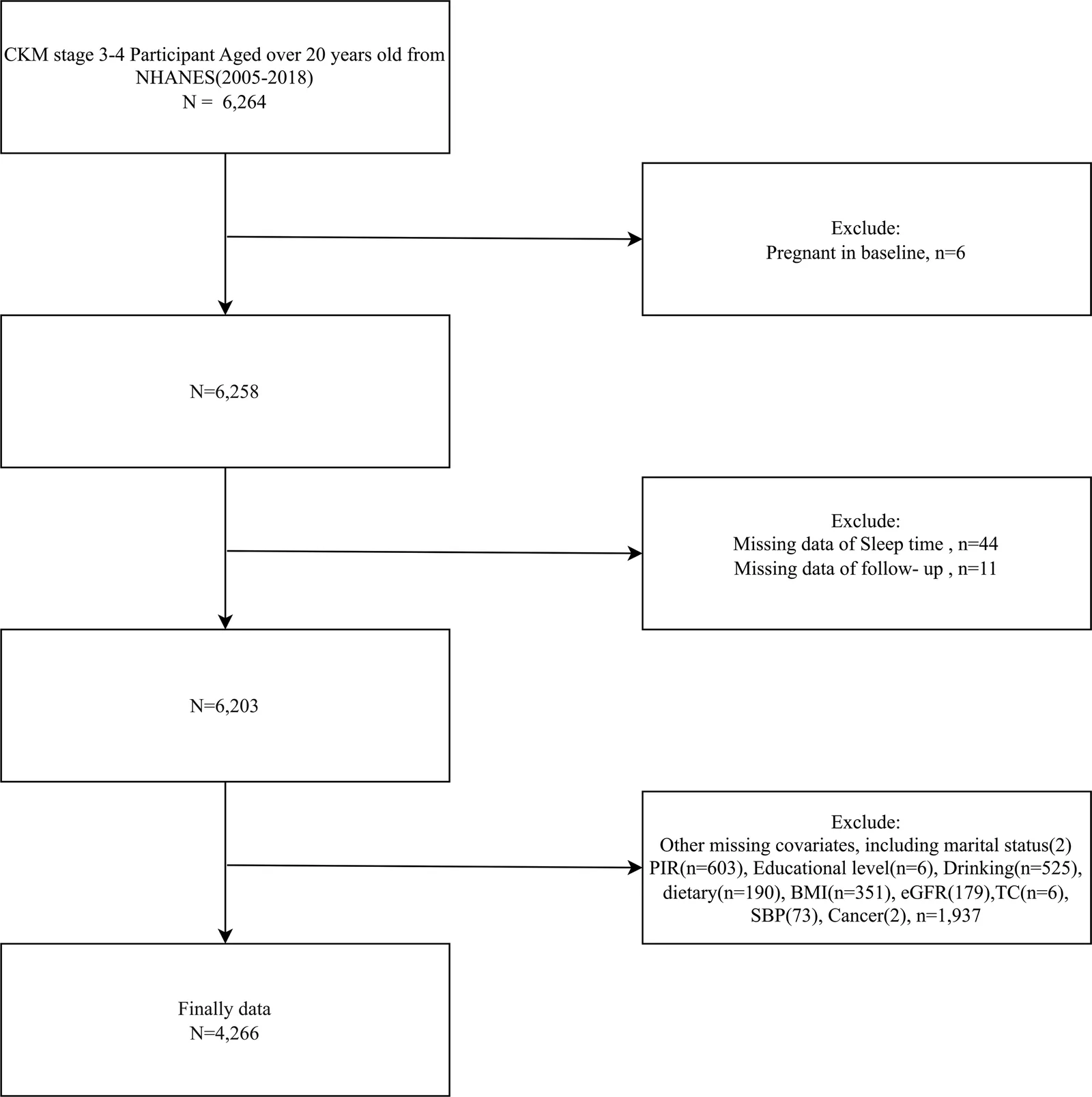
Flowchart of study subject selection.

### 2.2. Definition and diagnosis of advanced CKM syndrome

The present study classified the CKM syndrome stages into 5 distinct categories (0–4) as per the AHA framework.^[5]^ Advanced CKM syndrome consists mainly of Stages 3 and 4, used to differentiate patients’ cardiovascular risk or progress. Stages of ESRD are as follows: Stage 0: Subjects that do not have any identifiable cardiovascular, kidney, or other metabolic risk factors; Stage 1: Presence of risk factors which include excess total adiposity, abnormal fat distribution such as impaired glucose tolerance or prediabetes, or abdominal obesity; Stage 2: Diagnosis of high blood pressure, type 2 diabetes, hyperlipidemia, or kidney disease, which indicates substantially increased risks for the development of cardiorenal manifestations; Stage 3: Subjects with comorbidity of metabolic risk indicators, kidney disease or early high-risk cardiovascular phenotypes; Stage 4: Patients with clinical cardiovascular problems like heart failure or coronary heart disease. This stage is subdivided according to the existence of chronic renal insufficiency (4a) with no involvement of chronic kidney failure as well as (4b) with chronic renal failure.

### 2.3. Assessment of sleep time

The NHANES cycles collected sleep time data from 2005 to 2018 using a Computer-Assisted Personal Interview system. These participants all underwent a filled out a structured questionnaire reporting their usual night sleep hours within the last year. For the 2005 to 2014 cycles, sleep time was derived from the survey item asking participants about their typical nightly sleep hours on working days. Self-reported habitual nightly sleep hours were documented and analyzed as a continuous indicator. For the 2017 to 2018 cycles, weekday sleep time was estimated via 2 standardized questions regarding habitual bedtime and wake-up time. The actual sleeping hours were subsequently computed based on the time gap between the 2 reported time points.

Of note, sleep time was assessed using different methodologies across NHANES cycles: direct self-report (2005–2014) vs computed from bed/wake times (2017–2018). To account for this measurement heterogeneity, we performed stratified analyses by survey cycle (2005–2014 vs 2015–2018).For analysis, total sleep hours were classified into <7 hours, 7 to 8 hours (reference), as well as >8 hours.

### 2.4. Mortality outcome ascertainment

Using the baseline information sourced from the 2005 to 2018 NHANES cohorts regarding NDI until December 31, 2019, we derived the mortality data. The NDI matching was performed using a refined linkage algorithm based on the Fellegi-Sunter framework and adhered to public guidelines of the NCHS (https://www.cdc.gov/nchs/data-linkage/mortality-public.htm). The primary results were overall death rates and CVD fatal events. Death from any cause was referred to as all-cause mortality. Cardiovascular disease (CVD)-related death involved cerebrovascular diseases (I60–I69) and heart disease (ICD-10 codes I00–I09, I11, I13, I20–I51).

### 2.5. Covariates

NHANES offered extensive data on study participant variables as well as their physical assessment. The covariates and their processing standards were as follows:

#### 2.5.1. Demographics

Age, sex, educational level (Less than high school, High school, and above high school), and race/ethnicity (Mexican American, Other Hispanic, Non-Hispanic White, Non-Hispanic Black, or Other).

#### 2.5.2. Socioeconomic status

Socioeconomic status was assessed using the PIR, which was categorized into 3 grades: low income (<1.3), middle income (1.3–3.5), and high income (≥3.5).

#### 2.5.3. Lifestyle-related covariates

Alcohol consumption (Never, Former, and Current drinker); Smoking status (which is defined as Never, Former, as well as Current smoker); and Physical activity (Never, Moderate-intensity, as well as Vigorous-intensity physical activity).

#### 2.5.4. Dietary intake

Assessed using daily energy intake (kcal/day).

#### 2.5.5. Anthropometric and clinical indicators

Waist circumference (WC, cm); (BMI, kg/m^2^), classified into underweight (<18.5), healthy weight (18.5–24.9), overweight (25–29.9), or obese (≥30); Systolic/Diastolic blood pressure (SBP/DBP, mm Hg).

#### 2.5.6. Biochemical variables

Urinary albumin-to-creatinine ratio (UACR, mg/g); Triglycerides, mmol/L), Total cholesterol (TC, mmol/L), High-density lipoprotein cholesterol (HDL, mmol/L); and (eGFR, mL/min/1.73 m^2^), determined based on the 2021 CKD-EPI creatinine-based race-free equation.^[37]^

#### 2.5.7. Chronic disease diagnosis

* Hypertension^[38]^: Self-reported physician diagnosis, abnormal average blood pressure (SBP ≥ 140 mm Hg or DBP ≥ 90 mm Hg), or application of antihypertensive medication.

#### 2.5.8. *Diabetes*^[39]^

Self-reported physician diagnosis, HbA1c ≥ 6.5%, fasting plasma glucose ≥7.0 mmol/L, random plasma glucose ≥11.1 mmol/L, 2-hour oral glucose tolerance test glucose ≥11.1 mmol/L, or administration of glucose-lowering medication or insulin.

#### 2.5.9. Cancer status

Categorized as No or Yes.

#### 2.5.10. Cardiovascular disease (CVD)

Participants with prior diagnoses of coronary heart disease, myocardial infarction, congestive heart failure, angina, or stroke were classified into the CVD group.

### 2.6. Statistical analysis

Data analysis was carried out through SPSS version 27.0 as well as R version 4.5.0. The implementation of sampling weights, strata, and clusters was performed in all statistical analyses for NHANES data. The mean ± standard deviation was presented for normally distributed constant variables, and median (interquartile range, IQR) was presented for not normally distributed constant variables. The frequencies as well as proportions were used to describe categorical variables. Descriptive statistics were adopted for comparing baseline features of patients suffering from advanced CKM syndrome by sleep time groups. To assess the connection of sleep time with (all-cause and CVD) death, Kaplan–Meier survival curves were drawn, and variations across the categories were compared by performing the Log-rank test. Cox proportional hazards regression was adopted to obtain hazard ratios (HRs) and 95% confidence intervals (CIs) after adjusting for multiple confounding factors. To create 3 models, the researchers employed 2 different types of regressions. The details of these models included: Model 1: Unadjusted (crude model); Model 2: Adjusted for age, sex, race, education level, as well as PIR; Model 3: Adjusted for smoking, alcohol consumption, physical activity, energy intake, BMI, WC, SBP, DBP, eGFR, UACR, TC, HDL, diabetes, hypertension, and cancer. To clarify the causal assumptions underlying covariate selection, we constructed a Directed Acyclic Graph illustrating the hypothesized relationships between sleep time, confounders, mediators, and mortality outcomes (Supplementary Figure 1, Supplemental Digital Content 2). In this framework, demographic variables (age, sex, race, education, PIR) were treated as confounders, while cardiorenal-metabolic variables (eGFR, diabetes, hypertension, BMI, lipids, UACR) were considered potential mediators on the causal pathway. To explore possible nonlinear relationships, they relied on a multivariable-adjusted restricted cubic spline (RCS) Cox regression model. Estimation of threshold points was carried out along with trend analysis in case of nonlinearity. Moreover, subordinate analyses (age, sex, etc stratifications analyses) and sensitivity analyses (using varying adjustment strategies and/or removing a potential confounder) were conducted to assess the result robustness. All statistical tests were performed bilaterally, with statistical significance defined as a *P*-value <.05.

#### 2.6.1. Frailty-adjusted analysis

To address potential reverse causality, we constructed a frailty index (FI) based on 49 health deficits available in NHANES. A FI-adjusted Cox model (Model 4) was built by adding the continuous FI as an additional covariate to Model 3. We also performed stratified analyses by frailty status (non-frail: FI < 0.21; frail: FI ≥ 0.21) to examine whether the sleep-mortality association differed by frailty level.

#### 2.6.2. Propensity score matching

To further reduce confounding, we performed 1:1 propensity score matching (PSM) between the long sleep (>8 hours) and reference (7–8 hours) groups. Matching variables included all Model 3 covariates plus the continuous FI, using nearest-neighbor matching with a caliper of 0.2 standard deviation of the logit of the propensity score. Cox regression was then repeated in the matched cohort.

#### 2.6.3. Multiple imputation

Multiple imputation (MI) was performed to handle missing covariate data. Missing values for demographic, lifestyle, and clinical variables were assumed to be missing at random (Supplementary Table S8, Supplemental Digital Content 3). We generated 5 imputed datasets using a fully conditional specification algorithm, which is suitable for mixed continuous and categorical variables. The imputation model included all baseline covariates, exposure variables, and outcome endpoints involved in the final analytical models to preserve statistical association information.

## 3. Results

### 3.1. Baseline features of study subjects

The present study included 4266 patients suffering from advanced CKM syndrome. In accordance with their own report on sleep time, they were subdivided into 3 categories: the 7 to 8 hours group (n = 2018), the <7 hours group (n = 1481), and the >8 hours group (n = 767). The features of the groups are presented in Table 1.

**Table 1:** Baseline features of advanced cardiovascular-kidney-metabolic syndrome patients in different sleep time groups based on weighted NHANES Data.

Variable		Sleep time			
	Total	7 to 8	<7	>8	*P-value*
n	**4266**	**2018**	**1481**	**767**	
N (weighted)	**19,647,277**	**9,809,509**	**6,057,605**	**3,780,164**	
Sleep time, h	7.23 ± 0.03	7.55 ± 0.01	5.36 ± 0.04	9.41 ± 0.04	<.0001
Age	67.49 ± 0.25	68.00 ± 0.35	64.76 ± 0.45	70.54 ± 0.37	<.0001
PIR	2.72 ± 0.04	2.87 ± 0.05	2.49 ± 0.07	2.68 ± 0.10	<.0001
Energy intake, kcal/d	1909.41 ± 20.47	1922.52 ± 24.98	1926.61 ± 44.28	1847.81 ± 35.99	.19
BMI	30.52 ± 0.14	30.26 ± 0.20	31.01 ± 0.25	30.41 ± 0.34	.066
WC, cm	106.88 ± 0.33	106.78 ± 0.48	107.42 ± 0.61	106.25 ± 0.84	.513
eGFR	73.72 ± 0.45	73.42 ± 0.64	76.65 ± 0.75	69.82 ± 1.17	<.0001
uACR, mg/g	119.67 ± 9.18	116.93 ± 14.64	132.60 ± 16.97	106.04 ± 16.00	.515
SBP, mm Hg	132.87 ± 0.45	132.76 ± 0.60	132.10 ± 0.77	134.39 ± 0.99	.194
DBP, mm Hg	67.99 ± 0.31	67.54 ± 0.41	69.47 ± 0.54	66.79 ± 0.72	.003
TG, mmol/L	2.00 ± 0.03	2.03 ± 0.04	2.01 ± 0.05	1.91 ± 0.06	.22
TC, mmol/L	4.79 ± 0.03	4.78 ± 0.03	4.83 ± 0.04	4.76 ± 0.06	.39
HDL, mmol/L	1.29 ± 0.01	1.28 ± 0.01	1.28 ± 0.02	1.33 ± 0.02	.053
Sex					.005
Male	2535 (56.31)	1234 (58.37)	887 (57.28)	414 (49.41)	
Female	1731 (43.69)	784 (41.63)	594 (42.72)	353 (50.59)	
Race					<.0001
Mexican American	479 (4.62)	239 (4.69)	149 (4.75)	91 (4.26)	
Non-Hispanic Black	953 (10.77)	368 (8.06)	451 (16.61)	134 (8.45)	
Non-Hispanic White	2273 (76.00)	1164 (79.81)	670 (68.48)	439 (78.17)	
Other Hispanic	314 (3.39)	134 (2.59)	122 (4.39)	58 (3.85)	
Other Race	247 (5.22)	113 (4.86)	89 (5.77)	45 (5.27)	
Education					.218
<high school	624 (8.47)	278 (7.78)	219 (8.87)	127 (9.63)	
High school	1805 (40.82)	831 (39.57)	658 (43.66)	316 (39.51)	
>High school	1837 (50.71)	909 (52.65)	604 (47.48)	324 (50.86)	
Smoke					<.001
Never	1476 (34.35)	670 (32.99)	488 (33.05)	318 (39.97)	
Former	1998 (47.85)	1000 (50.31)	664 (44.67)	334 (46.52)	
Now	792 (17.80)	348 (16.69)	329 (22.27)	115 (13.51)	
Drinking					.021
Never	558 (11.33)	252 (11.15)	193 (11.68)	113 (11.25)	
Former	1448 (31.02)	657 (28.57)	534 (35.41)	257 (30.38)	
Now	2260 (57.64)	1109 (60.28)	754 (52.92)	397 (58.37)	
Physical activity					.545
No	2632 (57.28)	1233 (56.92)	894 (56.29)	505 (60.05)	
Moderate	1024 (27.04)	504 (28.22)	355 (26.43)	165 (25.08)	
Vigorous	606 (15.59)	278 (14.86)	231 (17.27)	97 (14.87)	
Diabetes					.276
No	2173 (54.65)	1061 (55.96)	739 (52.15)	373 (55.25)	
Yes	2093 (45.35)	957 (44.04)	742 (47.85)	394 (44.75)	
Hypertension					.295
No	783 (20.78)	396 (22.18)	248 (19.71)	139 (18.85)	
Yes	3483 (79.22)	1622 (77.82)	1233 (80.29)	628 (81.15)	
Cancer					.311
No	3329 (74.94)	1566 (74.87)	1179 (76.59)	584 (72.51)	
Yes	937 (25.06)	452 (25.13)	302 (23.41)	183 (27.49)	
ALL-cause					.024
No	2972 (73.13)	1375 (72.41)	1027 (71.36)	570 (77.86)	
Yes	1294 (26.87)	643 (27.59)	454 (28.64)	197 (22.14)	
CVD-cause					.39
No	3801 (90.54)	1799 (91.00)	1313 (89.53)	689 (90.94)	
Yes	465 (9.46)	219 (9.00)	168 (10.47)	78 (9.06)	

Weighted mean ± standard deviation was adopted to describe continuous variables, while categorical data were presented as case numbers and corresponding percentages. Both percentage values and P values were calculated based on sample weights.

The analytical findings of all the data were computed following the weighted analysis proposed by the NHANES program to guarantee national representative results. In particular, sample weights were employed to adjust for the complex stratified multi-stage probability sampling formulation. The weighted n given by NHANES was used for weighting in accordance with the number of people estimated within the total U.S. population each sample represents; this would make it less susceptible to sampling bias errors. It is customary to establish the values of statistical indicators as weighted mean ± standard error (mean ± SE) or weighted percentages (%).

The subjects’ average age was 67.49 ± 0.25 years, which was highest in >8 hours group (70.54 ± 0.37 years) and lowest in <7 hours group (64.76 ± 0.45 years). Difference was statistically significant among all 3 groups *(P < .0001*). Variations were found in the PIR, eGFR, and UACR, too. Notably, the >8 hours group exhibited the lowest eGFR (69.82 ± 1.17, *P < .0001*).

Males contributed 56.31% of sex-wise distribution in population. The percentage of females in the groups that slept for >8 hours was significantly higher (50.59%, *P = .005*). There were pronounced variations in terms of racial distribution (*P < .0001*) in all the groups, with the predominant group being Non-Hispanic Whites (76%). However, the most remarkable finding was an increased proportion of Non-Hispanic Blacks in the <7 hours category (16.61%).

Smoking status varied significantly across the groups (*P < .001*). The group with sleep time under 7 hours had the largest proportion of active smokers, reaching 22.27%. Alcohol consumption also showed variation (*P = .021*), with the > 8 hours group having the lowest percentage of current drinkers (58.37%). No significant difference was identified in the physical activity (*P = .545*).

Generally, there existed no notable variations between the groups with respect to metabolic characteristics such as BMI and WC (BMI: *P = .066*; WC: *P = .513*). The groups’ TC, Triglycerides, and HDL-cholesterol serum biochemical markers did not exhibit significant differences (*P > .05*). The category with normal BMI showed no variation in (SBP) (*P = .194*) relative to the variable category, but DBP showed variation (*P = .003*).

Diabetes (*P = .276*), hypertension (*P = .295*), as well as cancer (*P = .311*) proportions did not vary across the groups. Analysis results revealed that all-cause mortality showed significant differences across sleep time categories. The *P*-value was .024. The >8 hours group had the lowest weighted mortality belonging to sleep time categories (22.14%). The <7 hours group had the highest weighted mortality belonging to all sleep time categories (28.64%). Mortality from CVD did not vary across the 3 categories (*P = .39*).

### 3.2. Connection between sleep time and advanced CKM death

Of these 4266 subjects included in the study, 1294 (26.87%) all-cause deaths and 465 (9.46%) cardiovascular deaths occurred after a median follow-up duration spanning 7.6 years (Interquartile Range [IQR]: 4.1–11.9 years).

Results from the Kaplan–Meier survival analysis (Figs. 2A–2B) showed that for overall and cardiovascular death, there existed significant differences in survival distributions amongst different time spent sleeping categories (Log-rank test, $*P* < .05$). The rate of survival for individuals with a sleep time exceeding 8 hours was remarkably lower compared to those who had 7 to 8 hours of sleep.

**Figure 2.**
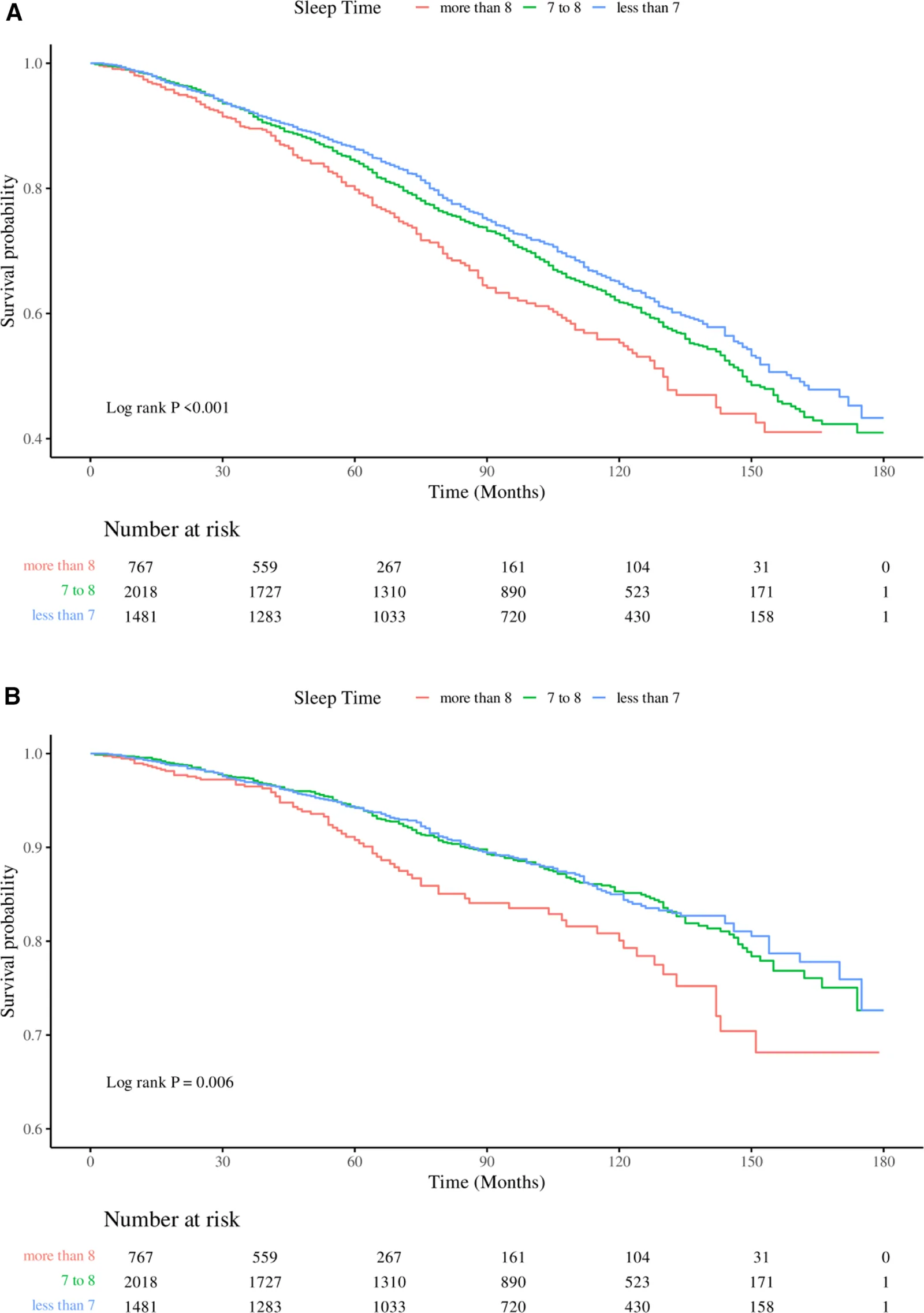
(A) Kaplan–Meier survival curves for all-cause death, (B) Kaplan–Meier survival curves for cardiovascular mortality.

In order to evaluate the connection between sleep time and risk for death, 3 multivariable Cox proportional hazards regression models were hereby established (Table 2. Model 1 served as a crude analysis model (not adjusted). Model 2 was modified for age, race/ethnicity, sex, educational level, and PIR. Model 3 (the completely adjusted one) further modified Model 2 in terms of smoking, alcohol intake, physical activity, energy intake, BMI, WC, SBP, DBP, eGFR, UACR, TC, HDL-cholesterol, diabetes, hypertension, and history of cancer.

**Table 2:** Connection between sleep time and all-cause and cardiovascular death: NHANES 2005 to 2018.

	Model 1 HR (95%CI)	*P*-value	Model 2 HR (95%CI)	*P*-value	Model 3 HR (95%CI)	*P*-value
All-cause mortality						
Sleep time	1.09 (1.03,1.14)	<.001	1.09 (1.04,1.14)	**<.001**	1.03 (0.99,1.08)	.14
Sleep time						
7–8	ref	ref	ref	ref	ref	ref
<7	0.95 (0.80,1.12)	.52	0.92 (0.78,1.08)	.31	1.00 (0.86,1.17)	.95
>8	1.35 (1.12,1.63)	.002	1.29 (1.07,1.56)	**.01**	1.21 (1.00,1.46)	**.05**
CVD mortality						
Sleep time	1.08 (1.02,1.15)	**.01**	1.09 (1.02,1.16)	**.01**	1.01 (0.95,1.08)	.69
Sleep time						
7–8	ref	ref	ref	ref	ref	ref
<7	1.06 (0.83,1.35)	.64	1.03 (0.80,1.33)	.82	1.18 (0.92,1.51)	.20
>8	1.72 (1.26,2.34)	**<.001**	1.66 (1.23,2.25)	**.001**	1.53 (1.14,2.07)	**.005**

Model 1: No adjustment.

Model 2: Modified for age, sex, race, education, PIR.

Model 3: Modified for age, race, sex, PIR, education, smoking, drinking, energy intake, physical activity, BMI, WC, SBP, DBP, eGFR, uACR, TC, diabetes, high‐density lipoprotein cholesterol, hypertension, cancer.

In the row “Sleep Time,” hazard ratios represent the risk associated with each 1-hour increase in sleep time. The reference group for the categorical analysis is the 7 to 8 hour sleep time group.

CVD, cardiovascular disease.

To address potential reverse causality, a FI-adjusted model (Model 4) was constructed. As shown in Table 3, after adjusting for cumulative health deficits, long sleep time (>8 hours) remained a significant risk factor for CVD mortality (HR: 1.49, 95% CI: 1.11–2.01, *P* = .008), although the effect size was slightly attenuated compared to Model 3.

**Table 3:** Cox proportional hazards ratios for mortality according to sleep time after additional adjustment for frailty index.

Outcome	Sleep group	Model 3 HR (95%CI), *P*	Model 4 (FI-adjusted) HR (95%CI), *P*
All-cause mortality	7–8 h	Ref	Ref
	<7 h	1.00 (0.86,1.17), 0.95	0.99 (0.85,1.16), 0.92
	>8 h	1.21 (1.00,1.46), 0.05	1.18 (0.98,1.43), 0.08
Cardiovascular mortality	7–8 h	Ref	Ref
	<7 h	1.18 (0.92,1.51), 0.20	1.16 (0.91,1.49), 0.23
	>8 h	1.53 (1.14,2.07), 0.005	1.49 (1.11,2.01), 0.008

#### 3.2.1. For All-cause death

Relative to patients who slept for 7 to 8 hours, the completely adjusted Model 3 demonstrated that sleep time of >8 hours was marginally tied to all-cause death (HR: 1.21, 95% CI: 1.00–1.46, *P = .05*). No significant relation was identified between sleep time <7 hours and overall fatal events (HR: 1.00, 95% CI: 0.86–1.17, *P = .95*).

#### 3.2.2. For cardiovascular mortality

There was a stronger association for cardiovascular mortality the analysis found. CVD mortality risk was remarkably increased in individuals with >8 hours of sleep time (HR: 1.53, 95% CI: 1.14–2.07, *P = .005*). Likewise, no remarkable association was identified between sleep time <7 hours and CVD mortality (HR: 1.18, 95% CI: 0.92–1.51, *P = .2*).

To conclude, these results indicate that among individuals suffering from advanced CKM syndrome, long sleep time (>8 hours) represents an independent risk factor for overall and CVD-related fatal events, with particularly strong association seen with CVD mortality.

### 3.3. Nonlinear connection between sleep time and advanced CKM death

This research implemented RCS within multivariable Cox regression models to further assess sleep time (continuous) and death risk in this study. These models were modified for the same confounding variables that were described in model 3 (Table 2). Nonlinear sleep time associations with overall and CVD-related death risks can be seen in Figures 3A–3B, respectively.

**Figure 3.**
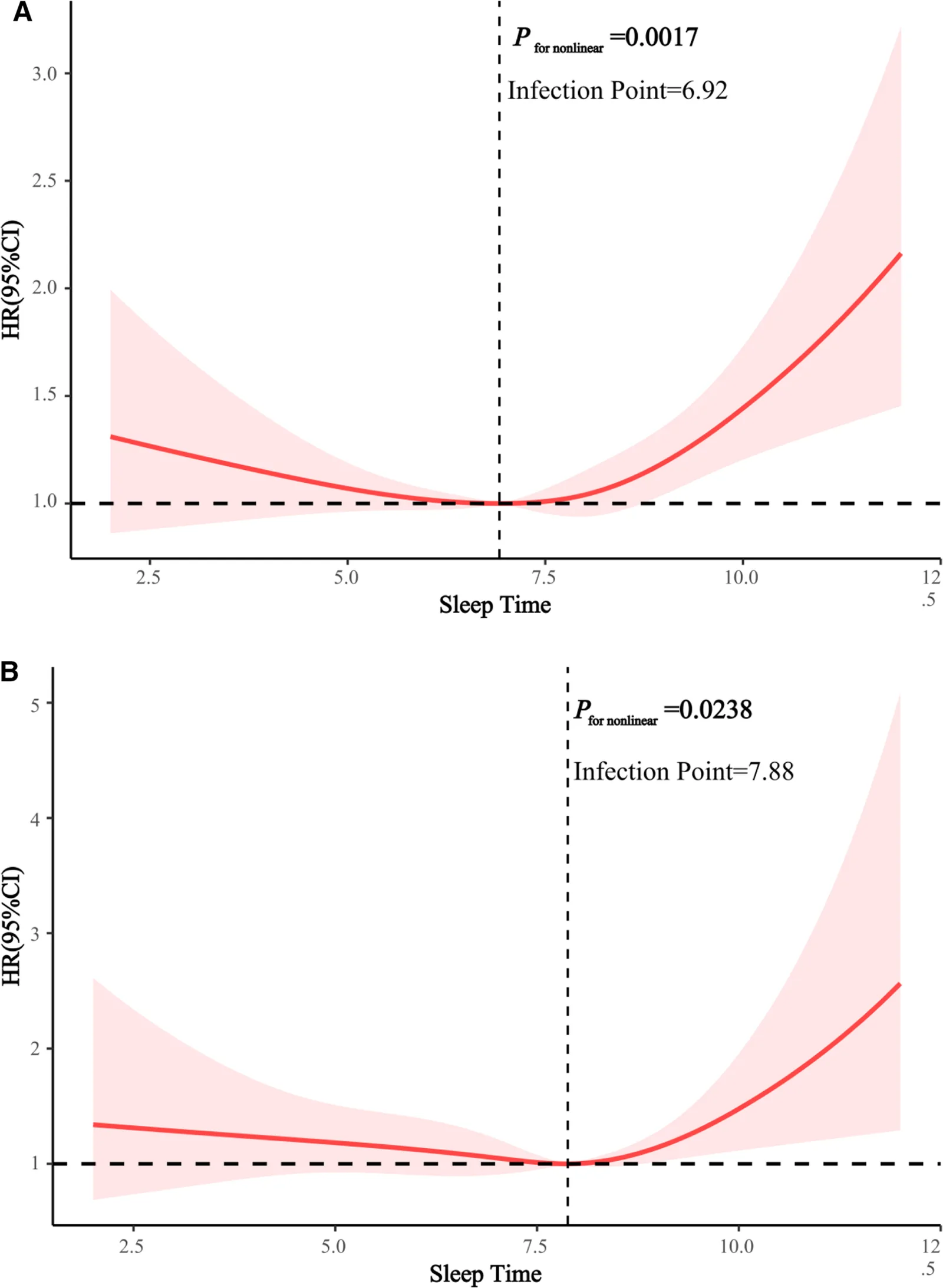
(A) Nonlinear connection between sleep time and all-cause death, (B) nonlinear connection between sleep time and cardiovascular mortality.

#### 3.3.1. For All-cause death

A statistically significant nonlinear U-shaped relationship was detected between sleep time and all-cause death risk (*P for nonlinearity = .0017*) (Fig. 3A). The nadir (the point of minimum risk) was reached in relation to a sleep of 6.92 hours. Once the sleeping hour lengthened to more than 6.92 hours, the risk of death started increasing quite substantially, becoming significantly elevated as sleep time exceeded 8 hours.

#### 3.3.2. For cardiovascular death

The study results evidenced a significant J-shaped association between sleep time and CVD mortality risk (*P for nonlinearity = .0238*) (Fig. 3B). The lowest risk of CVD death was at a sleep time of 7.88 hours. The link between long sleep period and CVD death was stronger than all-cause mortality linking. In particular, when sleep time was 8 hours and above, the risk curve exploded, indicating excessive sleep may be a greater hazard to the heart health of this community.

The RCS analysis showed a nonlinear U or J shape connection between sleep time and overall as well as cardiovascular death cases, in support of the optimal sleep time hypothesis. Excessive sleep as well as insufficient sleep was tied to a higher risk of death. Nevertheless, sleeping for too long had a more pronounced association with dying of cardiovascular patients.

### 3.4. Subgroup stratification and sensitivity analyses

Subgroup and sensitivity analyses were conducted to enhance the robustness of study findings and improve their generalizability across diverse populations.

#### 3.4.1. Subgroup analyses

Stratified analyses were performed to evaluate the consistency of the association across different subgroups. The results for all-cause and cardiovascular mortality are detailed in Supplementary Table S2, Supplemental Digital Content 4 and Supplementary Table S3, Supplemental Digital Content 5, respectively. Furthermore, we conducted a specific stratified analysis by CKM Stage 3 and 4, which confirmed the robustness of our findings (Supplementary Table S7, Supplemental Digital Content 6).

Subgroup analyses were hereby carried out following advanced CKM stages (Stage 3 vs Stage 4), which used fully adjusted Cox regression models for each stratum (Figure 4A–4B). In CKM Stage 4, long sleep time (>8 hours) was tied to a significantly higher risk for all-cause death (HR: 1.30, 95% CI: 1.05–1.60, *P = .014*) and CVD mortality (HR: 1.62, 95% CI: 1.10–2.38, *P* = .014). Among Stage 3 CKM patients, the connection between long sleep time and increased CVD death remained pronounced (HR: 1.70, 95% CI: 1.02–2.82, *P = .041*), but its connection with all-cause fatal events exhibited no statistical significance (HR: 1.27, 95% CI: 0.91–1.77, *P = .168*).

**Figure 4.**
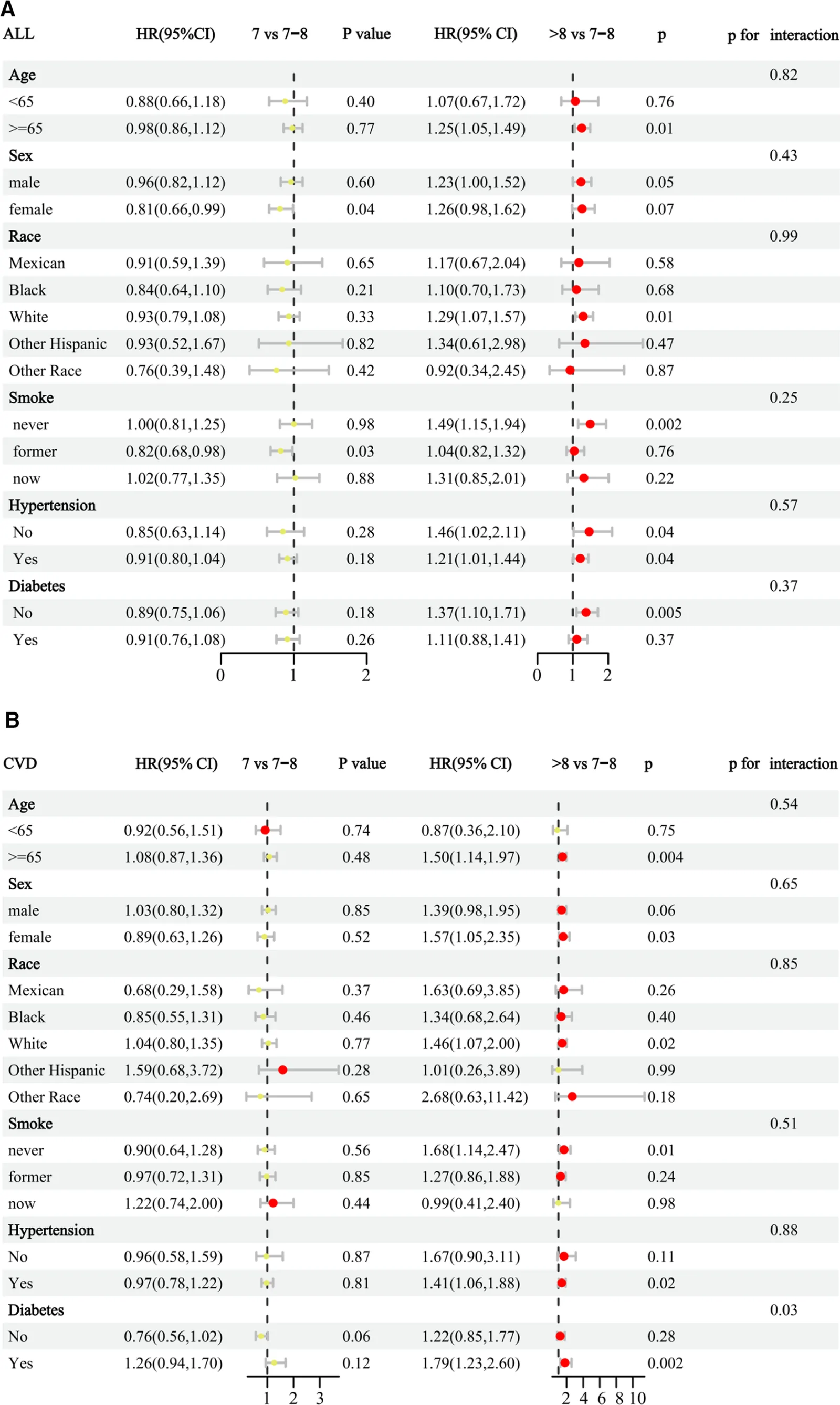
(A) Subgroup analyses of the association between sleep time and all-cause mortality. (B) Subgroup analyses of the connection between sleep time and cardiovascular death.

As suggested by the study results, long sleep time (>8 hours) was tied to cardiometabolic disease death cases among diabetics (HR: 1.79, 95% CI: 1.23–2.60, *P = .002*). Furthermore, the relationship between long sleep time and CVD death varied considerably based on diabetes status (*P for interaction = .03*).

#### 3.4.2. Sensitivity analyses

To assess the finding robustness, this research conducted sensitivity analyses with the 3 following assessments:

Those with a follow-up below 2 years were excluded to minimize reverse causality bias from early follow-up participants(Supplementary Table S5, Supplemental Digital Content 7). Likewise, our investigator showed that longer sleep time (>8 hours) still had a significant association with overall (HR: 1.27, 95% CI: 1.02–1.58, *P = .03*) and CVD disease death (HR: 1.56, 95% CI: 1.12–2.16, *P = .01*).

For the reason that malignancy may affect sleep as well as mortality risk simultaneously, participants with prior diagnosis of cancer at baseline were excluded (Supplementary Table S6, Supplemental Digital Content 8). The associations between long sleep time and the risks of all-cause death (HR: 1.26, 95% CI: 1.01–1.59, *P = .04*) and CVD death (HR: 1.51, 95% CI: 1.04–2.21, *P = .03*) in this subgroup remained unchanged.

Handling of Missing Data via MI: We used MI for the missing values of covariates (Supplementary Table S8, Supplemental Digital Content 3). Findings related to the imputed dataset demonstrate that the long sleep time with overall fatal events (HR: 1.22, 95% CI: 1.09–1.38, *P = .001*) and CVD death (HR: 1.28, 95% CI: 1.11–1.37, *P = .035*) were statistically significant.

In summary, findings from subgroup stratification and sensitivity verification demonstrated prolonged sleep time as an independent and stable risk factor for all-cause and cardiovascular death in individuals suffering from severe CKM syndrome.

#### 3.4.3. Frailty-adjusted analysis

After adjusting for the FI (Model 4), long sleep remained significantly associated with CVD mortality (HR: 1.49, 95% CI: 1.11–2.01, *P = .008*), though the HR was slightly attenuated compared to Model 3 (HR: 1.53). In stratified analysis (Table 4), long sleep conferred significantly increased CVD mortality risk in non-frail participants (HR: 1.72, 95% CI: 1.14–2.59, *P = .009*), but not in frail participants (HR: 1.33, 95% CI: 0.87–2.03, *P = .18*). The interaction between sleep time and frailty was not significant (*P for interaction = .29*).

**Table 4:** Association between sleep time and mortality stratified by frailty status.

Frailty stratum	Sleep time	All-cause Mortality HR (95%CI)	*P-value*	CVD Mortality HR (95%CI)	*P-value*
Non-frail (FI < 0.21)	7–8 h	Ref	–	Ref	–
	<7 h	1.03 (0.78,1.35)	0.82	1.10 (0.72,1.67)	0.65
	>8 h	1.31 (1.01,1.70)	0.04	1.72 (1.14,2.59)	0.009
Frail (FI ≥ 0.21)	7–8 h	Ref	–	Ref	–
	<7 h	0.95 (0.72,1.25)	0.72	1.11 (0.71,1.72)	0.64
	>8 h	1.11 (0.85,1.45)	0.43	1.33 (0.87,2.03)	0.18
*P* for interaction	–	0.33	–	0.29	–

#### 3.4.4. PSM

After 1:1 matching, baseline characteristics were well balanced between groups (all SMD < 0.1). In the matched cohort(Table 5), long sleep was associated with a borderline increased risk of all-cause mortality (HR: 1.24, 95% CI: 0.99–1.55, *P = .06*) and a significantly increased risk of CVD mortality (HR: 1.58, 95% CI: 1.13–2.21, *P = .007*).

**Table 5:** Association between long sleep time (>8 h) and mortality in the propensity score-matched cohort.

Outcome	Sleep group	Number of participants	Number of events	HR (95% CI)	*P-value*
All-cause mortality	7–8 h (reference)	692	207	Ref	–
	>8 h	692	231	1.24 (0.99, 1.55)	.06
Cardiovascular mortality	7–8 h (reference)	692	72	Ref	–
	>8 h	692	94	1.58 (1.13, 2.21)	.007

#### 3.4.5. Stratified analysis by survey cycle

When stratified by NHANES cycle, the association between long sleep and increased mortality risk was consistent across both periods (Supplementary Table S4, Supplemental Digital Content 9). For all-cause mortality, the HR for >8 hours sleep was 1.25 (95% CI: 1.03–1.52, *P = .022*) in 2005–2014 and 1.31 (95% CI: 1.06–1.77, *P = .007*) in 2015–2018 (*P for interaction = .567*). For CVD mortality, the corresponding HRs were 1.46 (95% CI: 1.07–1.98, *P = .016*) and 1.54 (95% CI: 1.02–2.56, *P = .009*), respectively (*P for interaction = .516*). These results suggest that the observed associations are robust to changes in sleep measurement methodology.

## 4. Discussion

### 4.1. Overview of the key findings

Sleep time is a modifiable lifestyle factor that has increasingly emerged as a public health issue for its strong association with various health problems as well as people’s quality of life. The present study makes the first attempt to investigate the connection between self-reported sleep time and the hazard of death in a high-risk cohort of individuals suffering from advanced CKM syndrome using the nationally representative NHANES (2005–2018) prospective cohort. In this population, we found that sleep time had a U-shaped connection with all-cause death, and a J-shaped connection with CVD fatal events. More specifically, long sleep time (>8 hours) was found to be a potent independent predicting factor of CVD death (HR: 1.53) after modification for confounding factors including demographics, lifestyle, comorbidities, and key laboratory indicators, such as eGFR and HbA1c. Note that the connection of long sleep time with all-cause death was close to being statistically significant, or simply, close to statistical significance. This study extended the U-shaped connection found in the general population or single disease cohorts, to the more pathophysiologically complex advanced CKM syndrome, offering unique clinical insights.

The American Heart Association’s “Life’s Essential 8” framework includes sleep as a component of your cardiovascular health.^[40]^ Our research further suggests an optimal sleep window of approximately 7 to 8 hours for individuals with advanced CKM syndrome, rather than precise cut-points. The RCS-derived nadirs (6.92 hours for all-cause mortality and 7.88 hours for CVD mortality) should be interpreted with caution given their wide uncertainty.

### 4.2. Nonlinear association between sleep time and mortality risk in advanced CKM

In comparison with previous studies focusing on general populations or individual conditions (e.g., isolated diabetes or hypertension), the U-shaped and J-shaped curves obtained in this study display similarities but also significant differences.

According to our study, long sleep time was found to be significantly more harmful for cardiovascular survival than short sleep time. Analysis of the baseline data (Table 1) suggested that long sleep did not directly cause death but rather was a surrogate of frailty or excessive chronic inflammatory burden or an undiagnosed condition (e.g., Obstructive Sleep Apnea [OSA]), reflecting severe potential comorbidities. This was especially true in the advanced CKM population. The long sleep group’s participants were older (mean age, 70.54 years) and had a lower level of eGFR. In advanced cases of CKM, poorly controlled congestive heart failure, moderate-to-severe sleep apnea-hypopnea syndrome, or chronic disease-related depression and fatigue might be included. These factors may worsen inflammatory responses, cause oxidative stress, and harm autonomic function, thus contributing to a vicious cycle with CKM syndrome responsible for raised mortality. The results of our study which used CKM model design is consistent with the analysis previous studies that longer sleep is associated with lower survival in patients with advanced heart failure or End Stage Renal Disease (ESRD).^[41]^

The RCS curves showed that short sleep time was connected with all-cause death; however, this association lost its significance after the Cox model covariate adjustments. This might evidence survivor bias or the complex metabolic cross-talk in advanced CKM syndrome. The buildup of metabolic waste caused by decreased renal function could interfere with the central nervous system in the patient with Stages 3 to 4 kidney disease that could fragment sleep. Thus, short sleep in this group might be a “symptom” of disease progression rather than a primary exposure. While short sleep itself did not have an independent association, its potential mechanisms including low-grade systemic inflammation and impaired glucose and lipid metabolism are still important and may be more evident in other populations or stages of disease.

### 4.3. Potential pathophysiological mechanisms

The link between sleep time and high mortality risk in advanced CKM syndrome patients may, through the following multidimensional mechanisms, intervene:

Systemic Chronic Inflammation and Oxidative Stress: CKM syndrome refers to a state of systemic inflammation triggered by obesity, insulin resistance, and chronic kidney injury. Studies show that abnormal sleep timing and having a long sleep time are positively associated with elevated pro-inflammatory cytokines (e.g., C-reactive protein, IL-6). Extended episodes of sleep can potentially aggravate inflammatory reactions owing to prolonged activation of the hypothalamic-pituitary-adrenal axis, resulting in endothelial dysfunction and expedited atherosclerosis.^[42]^

Circadian Misalignment and Metabolic Homeostasis: Both the kidneys and the heart have peripheral molecular clocks. In patients with Stage 4 CKM, a severe renal failure may disrupt the endogenous secretory pattern of melatonin. This circadian dysregulation not only affects sleep time but also interferes with sympathetic nervous system activity, resulting in blunted nocturnal blood pressure dipping (non-dipping). Thus, it increases the risk of acute cardiac events significantly.^[43]^

Cardiovascular Remodeling and Electrophysiological Stability: The cardiovascular remodeling and electrophysiological stability of CKM patients have long been recognized as a result of long sleep time characterized by a drastic fall in physical activity and increase in sedentary behavior. Moreover, excessive sleep may correlate with abnormal vagal tone and a lessened heart rate variability. CKM patients with preexisting myocardial hypertrophy or heart failure are easily predisposed to malignant arrhythmias.^[44]^

In addition to systemic inflammation, the accumulation of uremic toxins in advanced CKM syndrome plays a critical role in sleep disturbances and elevated mortality.^[45]^ In patients with advanced CKD (Stages 3–4), the impaired renal clearance leads to the retention of various uremic toxins, including parathyroid hormone (PTH), creatinine, urea, hippuric acid, and middle molecules such as interleukin-6 (IL-6), tumor necrosis factor-alpha (TNF-α), and beta-2 microglobulin (β2-MG).^[46]^ These substances are known to cross the blood-brain barrier or affect brain function indirectly, leading to uremic encephalopathy. This condition manifests as central nervous system depression, which clinically presents as excessive daytime somnolence and pathologically prolonged sleep time. Thus, the observed association between long sleep time and mortality may reflect a state of severe uremic toxicity and neurological impairment. These toxins not only disrupt sleep-wake cycles but also promote oxidative stress and endothelial damage, further accelerating cardiovascular deterioration.^[47]^ Unfortunately, measurements of these specific uremic toxins were not available in the NHANES dataset. Future studies incorporating direct measurements of uremic toxins are needed to elucidate the mechanistic pathways linking sleep time to mortality in this population.

### 4.4. Insights from subgroup and sensitivity analyses

Our subgroup findings show that disease staging may modify these associations, explained further in following. According to the findings, in patients with Stage 4 CKD, sleep time predictive value for survival was increased compared to that in Stage 3 CKD. In Stage 4 CKM (with established CVD or ESRD), the HR for overall and CVD death reached 1.30 and 1.62, respectively. It strongly indicated that as the involvement of heart, kidney, and metabolism damage entered the end stage, it affected the physiological homeostasis. Abnormal sleep patterns (especially excessive sleep) may be more sensitive markers of this systemic decompensation and heavier disease burden. In this context, duration of sleep has turned into a more sensitive survival warning sign compared to some standard indicators like cholesterol.^[48]^ On the contrary, short sleep time (<7 hours) did not present a pronounced association with mortality, which might be due to the disease states of this cohort or suggest that short and long sleep might involve different pathways.

Interestingly, diabetic status had a major effect on the association of hours of sleep with CVD death. In the subgroup of diabetes, long sleep time (>8 hours) had the most significant association with CVD death (HR: 1.79, 95% CI: 1.23–2.60, *P = .002*). In the diabetes subgroup, interaction testing confirmed diabetes remarkably modified this risk connection (*P for interaction = .03*). The excessive sleep in CKM patients with diabetes might signify a joint deeper metabolic derangement, chronic inflammatory responses, sleep-disordered breathing, or autonomic dysfunction. Thus, excessive sleeping may indicate a poorer baseline health or hidden cardiovascular complications. Hence, clinical follow-up and intervention should focus on this high-risk subgroup. The sleep time and screening for undetected sleep conditions are the most important clinical measures for reducing cardiovascular mortality in the diabetic patient.

We performed rigorous sensitivity analysis to ensure the robustness of our results. After eliminating early mortality to prevent reverse causality (more sleep as a pre-terminal symptom); excluding baseline cancer participants; and using MI for missing data, long sleep remained associated with increased mortality risk. The findings from this interpretation provide strong supports for the main conclusion that long sleep time is an independent, stable risk factor for mortality in patients with advanced CKM.

### 4.5. *Clinical implications and public health strategy* recommendations

Current management guidelines for CKM syndrome (such as the 2023 AHA guidelines) mainly focus on the role of drugs, such as SGLT2 inhibitors and GLP-1 receptor agonists.^[5]^ This study strongly recommends incorporating sleep health assessment into clinical pathways for chronic kidney disease management.

Individualized sleep monitoring for patients with advanced CKM who report sleeping more than 8 hours can be performed by PSG to identify occult conditions such as OSA.

Management of CKM should be multidisciplinary, involving cardiologists, nephrologists, and sleep specialists to implement early rehabilitation strategies for managing the frailty syndrome associated with long sleep time.

### 4.6. Strengths and limitations of the study

Strengths of the study included a large nationally representative sample with a long follow-up (2005–2018). The most up-to-date AHA definitions for CKM syndrome were applied strictly, allowing a more accurate stage disease differentiation. Additionally, we employed highly refined statistical models, which adjusted for an extensive range of confounders.

There were potential limitations in the study. First, the duration of sleep was assessed using self-report questionnaires. That could have resulted in recall bias or the possibility of misclassification when compared to objective recordings. Second, we did not have objective data on specific sleep disorders (e.g., sleep apnea), despite having adjusted for a wide range of confounders as well as for cancer and other comorbidities. There may, therefore, be residual confounding by unmeasured factors, such as detailed medication use, psychosocial support, or sleep-related factors. Moreover, it operated with baseline sleep questionnaires, without considering the dynamic aspect of sleep patterns over the observation period. Third, Model 3 adjusted for variables that may lie on the causal pathway between sleep time and mortality (e.g., eGFR, diabetes, hypertension, BMI). This overadjustment may have biased the estimates toward the null, potentially underestimating the total effect of sleep time. The attenuation of HRs from Model 2 (demographics only) to Model 3 suggests that part of the sleep-mortality association is mediated through these cardiorenal-metabolic pathways. Future studies employing mediation analysis are warranted to decompose the direct and indirect effects.Fourth, although we conducted frailty-adjusted analyses and PSM to mitigate reverse causality, residual confounding by unmeasured pre-terminal status cannot be entirely eliminated in observational studies. Long sleep may partly reflect underlying frailty or disease severity rather than being a purely modifiable risk factor. Fifth, sleep time was measured differently across NHANES cycles. Although stratified analyses yielded consistent results, the 2017 to 2018 data may capture time-in-bed rather than actual sleep time, introducing potential misclassification.Sixth, our study focused exclusively on sleep time and did not account for sleep quality or sleep disorders such as OSA, restless legs syndrome, or nocturia, which are highly prevalent in patients with advanced CKM syndrome and are independently associated with mortality. Although some sleep-related data are available in NHANES, we did not include them in the current analysis to maintain focus on sleep duration as the primary exposure. However, despite extensive adjustment for covariates, residual confounding from undiagnosed OSA or subclinical frailty cannot be entirely ruled out. Future studies integrating sleep quality and disorder measures are needed to comprehensively characterize the sleep-mortality relationship in this population. Without such data, our recommendation for polysomnography screening remains speculative and requires further validation.Ultimately, the generalizability of our findings is limited. The study population consisted primarily of Non-Hispanic White Americans (76%), and the results may not be directly applicable to non-US populations, individuals with earlier CKM stages (Stage 0–2), or racial/ethnic groups underrepresented in NHANES. Future studies in diverse international cohorts and across the full spectrum of CKM severity are needed to validate our findings.

## 5. Conclusion

In conclusion, this finding of a large prospective cohort study has shown that sleep time is a novel and independent predictor of all-cause and cardiovascular mortality in patients with advanced CKM syndrome. Our findings describe a nonlinear U-shaped or J-shaped association between sleep time and mortality risk, suggesting that 7 to 8 hours per night is the optimal sleep window for survival in this high-risk population.

Importantly, sleep time (>8 hours per night) increased the risk of all-cause as well as CVD mortality. As per the findings of this study, the sleep time in the clinical management of advanced CKM syndrome, should be utilized as an important health marker, which should readily be queried. In patients with consistent long sleep patterns, clinicians must maintain a high index of suspicion and screen and manage for severe comorbidities. The necessity of incorporating sleep monitoring into the future management guidelines of CKM is requested. Optimizing sleep health may be an important component of comprehensive management strategies in this complex high-risk population.

Future healthcare practices should not merely focus on the biochemical markers such as blood pressure, glucose, and renal function, but must incorporate healthy sleep as part of the routine management pathway of CKM syndrome. Interventions aimed at sleep time specifically intend to improve outcomes and quality of life in the long-term. In addition, to demonstrate the clinical merit of these interventions, prospective interventional trials with objective measures such as polysomnography or actigraphy are urgently required to ascertain whether modification of sleep patterns can translate into improved long-term survival for patients with advanced CKM syndrome.

## Author contributions

**Conceptualization:** Yangyang Xiong, Jiahua Liang.

**Formal analysis:** Yangyang Xiong, Jiahua Liang.

**Methodology:** Yangyang Xiong, Yue Yin.

**Project administration:** Yangyang Xiong.

**Resources:** Yangyang Xiong, Jing Wang, Ke Lan.

**Data curation:** Yue Yin, Jing Wang, Ke Lan.

**Investigation:** Yue Yin.

**Software:** Jing Wang, Ke Lan.

**Supervision:** Jing Wang.

**Validation:** Jing Wang.

**Visualization:** Wencong Han.

**Writing – original draft:** Yangyang Xiong, Yue Yin.

**Writing – review & editing:** Jiahua Liang, Wencong Han.


















