# Supplementary material for: Association of sleep time with all-cause and cardiovascular mortality among individuals with advanced cardiovascular-kidney-metabolic syndrome: A NHANES-based prospective cohort study
Source: Medicine (Baltimore). 2026 Oct 23;105(43):e51259. doi: 10.1097/MD.0000000000051259 (PMC13706387; doi:10.1097/MD.0000000000051259)
Supplement: Supplementary file 1 [file medi-105-e51259-s002.pdf]

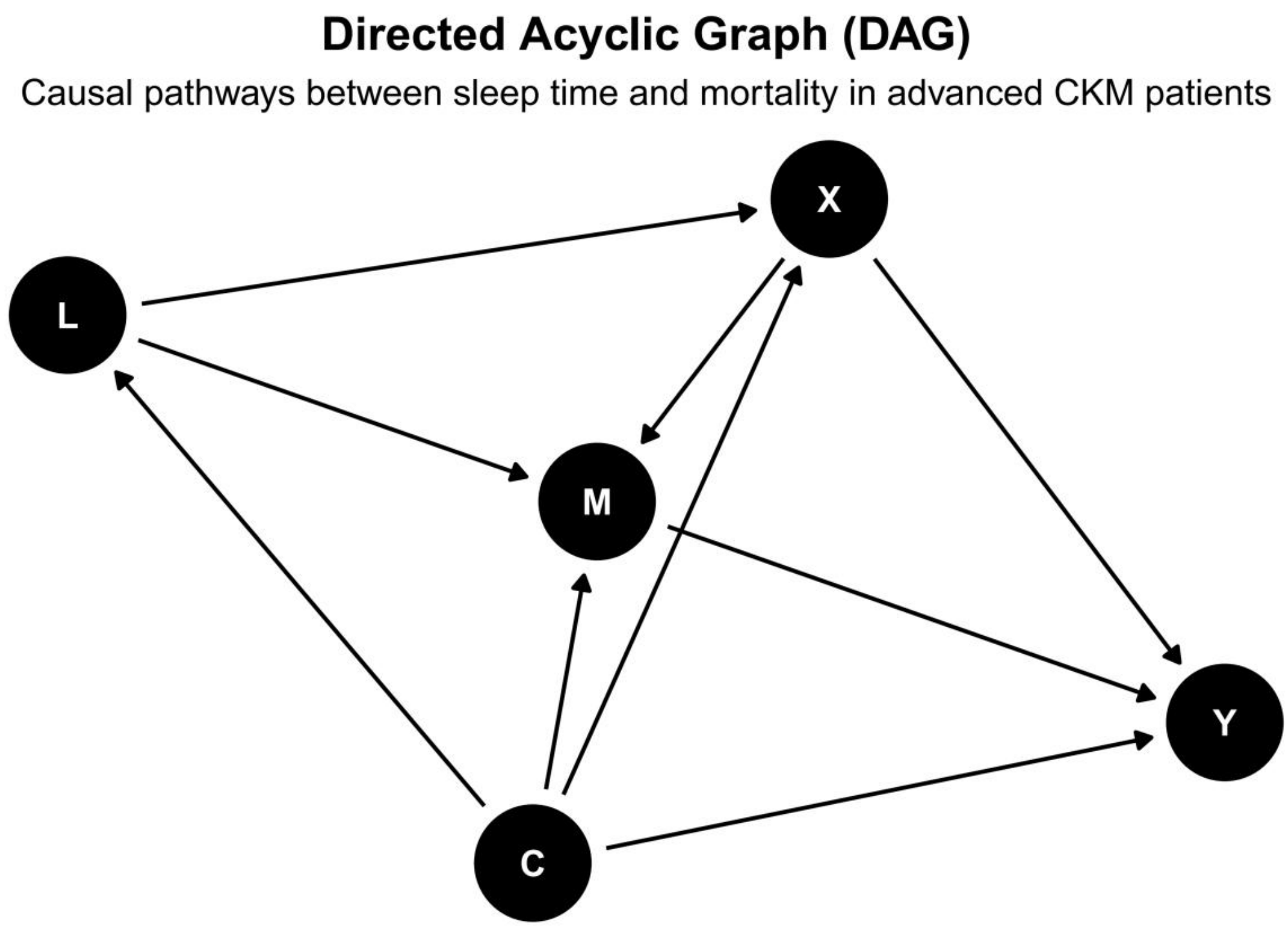

Supplementary Figure 1. Directed Acyclic Graph illustrating causal relationships between sleep time and mortality.

1. C: Demographic confounders (age, sex, race, education, poverty-income ratio; covariates for minimally adjusted Model 2).
2. L: Lifestyle covariates including smoking, alcohol, physical activity and cancer history.
3. X: Exposure, habitual self-reported sleep time.
4. M: Cardiorenal-metabolic mediators (BMI, hypertension, diabetes, eGFR, lipids, UACR) adjusted in fully adjusted Model 3.
5. Y: Primary outcomes (all-cause and cardiovascular mortality).

The pathway X → M → Y signifies the intermediate causal link. Adjustment for mediators M blocks this pathway and attenuates the total association between sleep time and mortality, leaving only the direct effect of sleep time on death risk.
