# Supplementary material for: Association of sleep time with all-cause and cardiovascular mortality among individuals with advanced cardiovascular-kidney-metabolic syndrome: A NHANES-based prospective cohort study
Source: Medicine (Baltimore). 2026 Oct 23;105(43):e51259. doi: 10.1097/MD.0000000000051259 (PMC13706387; doi:10.1097/MD.0000000000051259)
Supplement: Supplementary file 2 [file medi-105-e51259-s001.docx]

Supplementary Table S1. Baseline Features: Included participants vs Excluded participants with missing covariates

| **Variable** | **Included (n=4266)** | **Excluded (n=1937)** | ***P-value*** |
| --- | --- | --- | --- |
| Sleep time, hours | 7.23±0.03 | 7.19±0.06 | 0.541 |
| Age, years | 67.49±0.25 | 66.87±0.41 | 0.217 |
| PIR | 2.72±0.04 | 2.66±0.07 | 0.436 |
| Energy intake, kcal/day | 1909.41±20.47 | 1881.73±33.12 | 0.458 |
| BMI, kg/m² | 30.52±0.14 | 30.24±0.24 | 0.312 |
| WC, cm | 106.88±0.33 | 106.31±0.54 | 0.384 |
| eGFR, mL/min/1.73m² | 73.72±0.45 | 74.36±0.72 | 0.44 |
| uACR, mg/g | 119.67±9.18 | 126.34±14.21 | 0.685 |
| SBP, mmHg | 132.87±0.45 | 133.42±0.74 | 0.529 |
| DBP, mmHg | 67.99±0.31 | 68.35±0.50 | 0.543 |
| TG, mmol/L | 2.00±0.03 | 1.96±0.05 | 0.471 |
| TC, mmol/L | 4.79±0.03 | 4.76±0.05 | 0.582 |
| HDL, mmol/L | 1.29±0.01 | 1.30±0.02 | 0.61 |
| Sex |  |  | 0.163 |
| Male | 2535 (56.31%) | 1096 (53.94%) |  |
| Female | 1731 (43.69%) | 841 (46.06%) |  |
| Race/Ethnicity |  |  | 0.277 |
| Mexican American | 479 (4.62%) | 232 (5.11%) |  |
| Non-Hispanic Black | 953 (10.77%) | 457 (11.46%) |  |
| Non-Hispanic White | 2273 (76.00%) | 982 (74.13%) |  |
| Other Hispanic | 314 (3.39%) | 154 (3.90%) |  |
| Other Race | 247 (5.22%) | 112 (5.40%) |  |
| Education level |  |  | 0.35 |
| <High school | 624 (8.47%) | 297 (9.16%) |  |
| High school graduate | 1805 (40.82%) | 842 (42.31%) |  |
| >High school | 1837 (50.71%) | 798 (48.53%) |  |
| Smoking status |  |  | 0.225 |
| Never smoker | 1476 (34.35%) | 664 (32.47%) |  |
| Former smoker | 1998 (47.85%) | 920 (49.33%) |  |
| Current smoker | 792 (17.80%) | 353 (18.20%) |  |
| Alcohol drinking status |  |  | 0.406 |
| Never drinker | 558 (11.33%) | 268 (12.14%) |  |
| Former drinker | 1448 (31.02%) | 805 (32.51%) |  |
| Current drinker | 2260 (57.64%) | 864 (55.35%) |  |
| Physical activity |  |  | 0.328 |
| No activity | 2632 (57.28%) | 1187 (59.41%) |  |
| Moderate activity | 1024 (27.04%) | 446 (25.72%) |  |
| Vigorous activity | 606 (15.59%) | 304 (14.87%) |  |
| Diabetes |  |  | 0.472 |
| No | 2173 (54.65%) | 969 (53.24%) |  |
| Yes | 2093 (45.35%) | 968 (46.76%) |  |
| Hypertension |  |  | 0.516 |
| No | 783 (20.78%) | 402 (21.83%) |  |
| Yes | 3483 (79.22%) | 1535 (78.17%) |  |
| Cancer history |  |  | 0.364 |
| No | 3329 (74.94%) | 1486 (73.26%) |  |
| Yes | 937 (25.06%) | 451 (26.74%) |  |
| All-cause mortality |  |  | 0.573 |
| No event | 2972 (73.13%) | 1341 (74.26%) |  |
| Death | 1294 (26.87%) | 596 (25.74%) |  |
| CVD mortality |  |  | 0.491 |
| No event | 3801 (90.54%) | 1722 (91.31%) |  |
| CVD death | 465 (9.46%) | 215 (8.69%) |  |
