# Supplementary material for: Association of sleep time with all-cause and cardiovascular mortality among individuals with advanced cardiovascular-kidney-metabolic syndrome: A NHANES-based prospective cohort study
Source: Medicine (Baltimore). 2026 Oct 23;105(43):e51259. doi: 10.1097/MD.0000000000051259 (PMC13706387; doi:10.1097/MD.0000000000051259)
Supplement: Supplementary file 3 [file medi-105-e51259-s003.docx]

Supplementary Table S8.Multiple Imputation

**ALL：**

|  | **Model 1**  **HR (95%CI)** | ***P* value** | **Model 2**  **HR (95%CI)** | ***P* value** | **Model 3**  **HR (95%CI)** | ***P* value** |
| --- | --- | --- | --- | --- | --- | --- |
| **Sleep Time** | 1.10(1.07,1.13) | <0.0001 | 1.11 (1.08,1.14) | <0.001 | 1.02 (0.95,1.08) | 0.644 |
| **Sleep Time** |  |  |  |  |  |  |
| 7-8 | ref |  |  |  |  |  |
| <7 | 1.04 (0.92,1.17) | 0.578 | 0.98 (0.87,1.11) | 0.769 | 1.01 (0.89,1.14) | 0.907 |
| >8 | 1.36 (1.21,1.53) | <0.0001 | 1.33 (1.18,1.49) | <0.001 | 1.22 (1.09,1.38) | 0.001 |

Model 1: No-adjust

Model 2: adjusted for age, sex, race, education, PIR

Model 3: adjusted for age, sex, race, education, PIR, smoking, drinking, physical activity, energy intake, BMI, WC, SBP, DBP, eGFR, uACR, total cholesterol, high‐density lipoprotein cholesterol, Diabetes, Hypertension

**CVD：**

|  | **Model 1**  **HR (95%CI)** | ***P* value** | **Model 2**  **HR (95%CI)** | ***P* value** | **Model 3**  **HR (95%CI)** | ***P* value** |
| --- | --- | --- | --- | --- | --- | --- |
| **Sleep Time** | 1.05(0.97,1.15) | <0.001 | 1.11 (1.06,1.15) | <0.001 | 0.94 (0.89,1.00) | 0.050 |
| **Sleep Time** |  |  |  |  |  |  |
| 7-8 | ref |  |  |  |  |  |
| <7 | 1.02 (0.83,1.25) | 0.861 | 0.97 (0.79,1.19) | 0.768 | 1.00 (0.81,1.23) | 0.974 |
| >8 | 1.31 (1.08,1.59) | 0.006 | 1.29  (1.06,1.57) | 0.011 | 1.28  (1.11,1.37) | 0.035 |

Model 1: No-adjust

Model 2: adjusted for age, sex, race, education, PIR

Model 3: adjusted for age, sex, race, education, PIR, smoking, drinking, physical activity, energy intake, BMI, WC, SBP, DBP, eGFR, uACR, total cholesterol, high‐density lipoprotein cholesterol, Diabetes, Hypertension
