# Supplementary material for: Association of sleep time with all-cause and cardiovascular mortality among individuals with advanced cardiovascular-kidney-metabolic syndrome: A NHANES-based prospective cohort study
Source: Medicine (Baltimore). 2026 Oct 23;105(43):e51259. doi: 10.1097/MD.0000000000051259 (PMC13706387; doi:10.1097/MD.0000000000051259)
Supplement: Supplementary file 4 [file medi-105-e51259-s004.docx]

| Supplementary Table S2. Stratified analysis of the association between sleep time and all-cause mortality. | | | | | | |
| --- | --- | --- | --- | --- | --- | --- |
| **Stratifying Factor** | **Subgroup Stratum** | **<7 h vs. 7–8 h HR (95% CI)** | ***P*** | **>8 h vs. 7–8 h HR (95% CI)** | ***P*** | ***P_interaction*** |
| Age | <65 years | 0.88 (0.66, 1.18) | 0.4 | 1.07 (0.67, 1.72) | 0.76 | 0.82 |
|  | ≥65 years | 0.98 (0.86, 1.12) | 0.77 | 1.25 (1.05, 1.49) | 0.01 |  |
| Sex | Male | 0.96 (0.82, 1.12) | 0.6 | 1.23 (1.00, 1.52) | 0.05 | 0.43 |
|  | Female | 0.81 (0.66, 0.99) | 0.04 | 1.26 (0.98, 1.62) | 0.07 |  |
| Race/Ethnicity | Mexican American | 0.91 (0.59, 1.39) | 0.65 | 1.17 (0.67, 2.04) | 0.58 | 0.99 |
|  | Non-Hispanic Black | 0.84 (0.64, 1.10) | 0.21 | 1.10 (0.70, 1.73) | 0.68 |  |
|  | Non-Hispanic White | 0.93 (0.79, 1.08) | 0.33 | 1.29 (1.07, 1.57) | 0.01 |  |
|  | Other Hispanic | 0.93 (0.52, 1.67) | 0.82 | 1.34 (0.61, 2.98) | 0.47 |  |
|  | Other Race | 0.76 (0.39, 1.48) | 0.42 | 0.92 (0.34, 2.45) | 0.87 |  |
| Education Level | Less than high school | 0.94 (0.73, 1.21) | 0.63 | 1.24 (0.96, 1.60) | 0.09 | 0.71 |
|  | High school | 0.90 (0.76, 1.07) | 0.24 | 1.22 (1.01, 1.47) | 0.04 |  |
|  | Above high school | 0.97 (0.83, 1.13) | 0.7 | 1.19 (0.98, 1.44) | 0.07 |  |
| Poverty-Income Ratio (PIR) | Low income (<1.3) | 0.92 (0.71, 1.19) | 0.52 | 1.28 (0.99, 1.65) | 0.06 | 0.66 |
|  | Middle income (1.3–3.5) | 0.91 (0.77, 1.08) | 0.28 | 1.23 (1.02, 1.48) | 0.03 |  |
|  | High income (≥3.5) | 0.95 (0.80, 1.13) | 0.57 | 1.17 (0.95, 1.43) | 0.13 |  |
| Smoking Status | Never smoker | 1.00 (0.81, 1.25) | 0.98 | 1.49 (1.15, 1.94) | 0.002 | 0.25 |
|  | Former smoker | 0.82 (0.68, 0.98) | 0.03 | 1.04 (0.82, 1.32) | 0.76 |  |
|  | Current smoker | 1.02 (0.77, 1.35) | 0.88 | 1.31 (0.85, 2.01) | 0.22 |  |
| Alcohol Consumption | Never drinker | 0.93 (0.70, 1.23) | 0.62 | 1.30 (0.97, 1.74) | 0.08 | 0.78 |
|  | Former drinker | 0.89 (0.73, 1.08) | 0.24 | 1.21 (0.98, 1.49) | 0.07 |  |
|  | Current drinker | 0.95 (0.81, 1.11) | 0.53 | 1.20 (0.99, 1.45) | 0.06 |  |
| Physical Activity | No activity | 0.94 (0.80, 1.10) | 0.44 | 1.26 (1.05, 1.51) | 0.01 | 0.84 |
|  | Moderate activity | 0.88 (0.71, 1.09) | 0.25 | 1.18 (0.93, 1.49) | 0.16 |  |
|  | Vigorous activity | 0.91 (0.68, 1.22) | 0.53 | 1.15 (0.84, 1.57) | 0.38 |  |
| BMI Category | Underweight (<18.5) | 0.87 (0.54, 1.40) | 0.56 | 1.32 (0.78, 2.24) | 0.3 | 0.91 |
|  | Normal weight (18.5–24.9) | 0.92 (0.74, 1.14) | 0.45 | 1.24 (0.99, 1.55) | 0.06 |  |
|  | Overweight (25–29.9) | 0.93 (0.76, 1.14) | 0.49 | 1.22 (0.98, 1.52) | 0.07 |  |
|  | Obese (≥30) | 0.94 (0.79, 1.12) | 0.49 | 1.19 (0.97, 1.46) | 0.09 |  |
| Hypertension | No hypertension | 0.85 (0.63, 1.14) | 0.28 | 1.46 (1.02, 2.11) | 0.04 | 0.57 |
|  | With hypertension | 0.91 (0.80, 1.04) | 0.18 | 1.21 (1.01, 1.44) | 0.04 |  |
| Diabetes | No diabetes | 0.89 (0.75, 1.06) | 0.18 | 1.37 (1.10, 1.71) | 0.005 | 0.37 |
|  | With diabetes | 0.91 (0.76, 1.08) | 0.26 | 1.11 (0.88, 1.41) | 0.37 |  |
| Cancer History | No cancer | 0.92 (0.79, 1.07) | 0.29 | 1.23 (1.02, 1.48) | 0.03 | 0.74 |
|  | With cancer | 0.96 (0.75, 1.25) | 0.77 | 1.18 (0.89, 1.57) | 0.25 |  |
