# Supplementary material for: Association of sleep time with all-cause and cardiovascular mortality among individuals with advanced cardiovascular-kidney-metabolic syndrome: A NHANES-based prospective cohort study
Source: Medicine (Baltimore). 2026 Oct 23;105(43):e51259. doi: 10.1097/MD.0000000000051259 (PMC13706387; doi:10.1097/MD.0000000000051259)
Supplement: Supplementary file 5 [file medi-105-e51259-s005.docx]

Supplementary Table S3. Stratified analysis of the association between sleep time and cardiovascular mortality.

| **Stratifying Factor** | **Subgroup Stratum** | **<7 h vs. 7–8 h HR (95% CI)** | ***P*** | **>8 h vs. 7–8 h HR (95% CI)** | ***P*** | ***P_interaction*** |
| --- | --- | --- | --- | --- | --- | --- |
| Age | <65 years | 0.92 (0.56, 1.51) | 0.74 | 0.87 (0.36, 2.10) | 0.75 | 0.54 |
|  | ≥65 years | 1.08 (0.87, 1.36) | 0.48 | 1.50 (1.14, 1.97) | 0.004 |  |
| Sex | Male | 1.03 (0.80, 1.32) | 0.85 | 1.39 (0.98, 1.95) | 0.06 | 0.65 |
|  | Female | 0.89 (0.63, 1.26) | 0.52 | 1.57 (1.05, 2.35) | 0.03 |  |
| Race/Ethnicity | Mexican American | 0.68 (0.29, 1.58) | 0.37 | 1.63 (0.69, 3.85) | 0.26 | 0.85 |
|  | Non-Hispanic Black | 0.85 (0.55, 1.31) | 0.46 | 1.34 (0.68, 2.64) | 0.4 |  |
|  | Non-Hispanic White | 1.04 (0.80, 1.35) | 0.77 | 1.46 (1.07, 2.00) | 0.02 |  |
|  | Other Hispanic | 1.59 (0.68, 3.72) | 0.28 | 1.01 (0.26, 3.89) | 0.99 |  |
|  | Other Race | 0.74 (0.20, 2.69) | 0.65 | 2.68 (0.63, 11.42) | 0.18 |  |
| Education Level | Less than high school | 0.97 (0.62, 1.52) | 0.89 | 1.61 (1.08, 2.40) | 0.02 | 0.76 |
|  | High school | 1.02 (0.74, 1.41) | 0.91 | 1.48 (1.06, 2.08) | 0.02 |  |
|  | Above high school | 0.93 (0.67, 1.29) | 0.66 | 1.36 (0.97, 1.91) | 0.07 |  |
| Poverty-Income Ratio (PIR) | Low income (<1.3) | 1.05 (0.66, 1.67) | 0.84 | 1.64 (1.07, 2.51) | 0.02 | 0.61 |
|  | Middle income (1.3–3.5) | 0.99 (0.72, 1.34) | 0.94 | 1.47 (1.06, 2.04) | 0.02 |  |
|  | High income (≥3.5) | 0.91 (0.64, 1.29) | 0.6 | 1.33 (0.92, 1.92) | 0.13 |  |
| Smoking Status | Never smoker | 0.90 (0.64, 1.28) | 0.56 | 1.68 (1.14, 2.47) | 0.01 | 0.51 |
|  | Former smoker | 0.97 (0.72, 1.31) | 0.85 | 1.27 (0.70, 1.88) | 0.24 |  |
|  | Current smoker | 1.22 (0.74, 2.00) | 0.44 | 0.99 (0.41, 2.40) | 0.98 |  |
| Alcohol Consumption | Never drinker | 1.04 (0.64, 1.69) | 0.87 | 1.59 (1.01, 2.51) | 0.04 | 0.81 |
|  | Former drinker | 1.01 (0.71, 1.44) | 0.96 | 1.45 (1.01, 2.08) | 0.04 |  |
|  | Current drinker | 0.94 (0.69, 1.29) | 0.71 | 1.38 (0.99, 1.93) | 0.06 |  |
| Physical Activity | No activity | 1.03 (0.78, 1.36) | 0.84 | 1.51 (1.12, 2.05) | 0.007 | 0.88 |
|  | Moderate activity | 0.95 (0.65, 1.39) | 0.79 | 1.37 (0.92, 2.04) | 0.12 |  |
|  | Vigorous activity | 0.88 (0.52, 1.48) | 0.63 | 1.26 (0.73, 2.18) | 0.4 |  |
| BMI Category | Underweight (<18.5) | 0.83 (0.36, 1.92) | 0.66 | 1.72 (0.82, 3.60) | 0.15 | 0.94 |
|  | Normal weight (18.5–24.9) | 0.95 (0.65, 1.39) | 0.79 | 1.52 (1.05, 2.20) | 0.03 |  |
|  | Overweight (25–29.9) | 1.01 (0.70, 1.45) | 0.96 | 1.44 (1.01, 2.06) | 0.04 |  |
|  | Obese (≥30) | 1.04 (0.74, 1.46) | 0.83 | 1.36 (0.96, 1.93) | 0.08 |  |
| Hypertension | No hypertension | 0.96 (0.58, 1.59) | 0.87 | 1.67 (0.90, 3.11) | 0.11 | 0.88 |
|  | With hypertension | 0.97 (0.78, 1.22) | 0.81 | 1.41 (1.06, 1.88) | 0.02 |  |
| Diabetes | No diabetes | 0.76 (0.56, 1.02) | 0.06 | 1.22 (0.85, 1.77) | 0.28 | 0.03 |
|  | With diabetes | 1.26 (0.94, 1.70) | 0.12 | 1.79 (1.23, 2.60) | 0.002 |  |
| Cancer History | No cancer | 0.98 (0.76, 1.26) | 0.86 | 1.46 (1.08, 1.98) | 0.01 | 0.79 |
|  | With cancer | 1.05 (0.70, 1.58) | 0.81 | 1.32 (0.86, 1.98) | 0.2 |  |
