# Supplementary material for: Association of sleep time with all-cause and cardiovascular mortality among individuals with advanced cardiovascular-kidney-metabolic syndrome: A NHANES-based prospective cohort study
Source: Medicine (Baltimore). 2026 Oct 23;105(43):e51259. doi: 10.1097/MD.0000000000051259 (PMC13706387; doi:10.1097/MD.0000000000051259)
Supplement: Supplementary file 6 [file medi-105-e51259-s006.docx]

Supplementary Table S7.Stratified Analysis by CKM Stage 3-4

| **CKM3** | **ALL**  **Model**  **HR (95%CI)** | ***P* value** | **CVD**  **Model**  **HR (95%CI)** | ***P* value** |
| --- | --- | --- | --- | --- |
| **Sleep Time** | 1.031(0.938,1.133) | 0.533 | 0.969(0.898,1.045) | 0.409 |
| **Sleep Time** |  |  |  |  |
| 7-8 | Ref. | Ref. | Ref. | Ref. |
| <7 | 0.997(0.782,1.269) | 0.978 | 1.009(0.639,1.593) | 0.969 |
| >8 | 1.267(0.905,1.773) | 0.168 | 1.697(1.022,2.818) | **0.041** |
| **CKM4** |  |  |  |  |
| **Sleep Time** | 1.032(0.979,1.088) | 0.243 | 0.969(0.898,1.045) | 0.409 |
| **Sleep Time** |  |  |  |  |
| 7-8 | Ref. | Ref. | Ref. | Ref. |
| <7 | 0.870(0.705,1.073) | 0.193 | 1.032(0.741,1.436) | 0.853 |
| >8 | 1.297(1.053,1.597) | **0.014** | 1.619(1.103,2.375) | **0.014** |

Model: adjusted for age, sex, race, education, PIR, smoking, drinking, physical activity, energy intake, BMI, WC, SBP, DBP, eGFR, uACR, total cholesterol, high‐density lipoprotein cholesterol, Diabetes, Hypertension
