# Supplementary material for: Association of sleep time with all-cause and cardiovascular mortality among individuals with advanced cardiovascular-kidney-metabolic syndrome: A NHANES-based prospective cohort study
Source: Medicine (Baltimore). 2026 Oct 23;105(43):e51259. doi: 10.1097/MD.0000000000051259 (PMC13706387; doi:10.1097/MD.0000000000051259)
Supplement: Supplementary file 7 [file medi-105-e51259-s007.docx]

Supplementary Table S5.Excluding Patients with Less Than Two Years of Follow-up

**ALL：**

|  | **Model 1**  **HR (95%CI)** | ***P* value** | **Model 2**  **HR (95%CI)** | ***P* value** | **Model 3**  **HR (95%CI)** | ***P* value** |
| --- | --- | --- | --- | --- | --- | --- |
| **Sleep Time** | 1.09(1.04,1.15) | **0.001** | 1.09(1.04,1.15) | **<0.001** | 1.08(1.03,1.14) | **0.003** |
| **Sleep Time** |  |  |  |  |  |  |
| 7-8 | ref | ref | ref | ref | ref | ref |
| <7 | 0.89(0.75,1.07) | 0.23 | 0.88(0.74,1.04) | 0.13 | 0.89(0.75,1.06) | 0.20 |
| >8 | 1.33(1.08,1.64) | **0.01** | 1.28(1.03,1.57) | **0.02** | 1.27(1.02,1.58) | **0.03** |

Model 1: No-adjust

Model 2: adjusted for age, sex, race, education, PIR

Model 3: adjusted for age, sex, race, education, PIR, smoking, drinking, physical activity, energy intake, BMI, WC, SBP, DBP, eGFR, uACR, total cholesterol, high‐density lipoprotein cholesterol, Diabetes, Hypertension, Cancer

**CVD：**

|  | **Model 1**  **HR (95%CI)** | ***P* value** | **Model 2**  **HR (95%CI)** | ***P* value** | **Model 3**  **HR (95%CI)** | ***P* value** |
| --- | --- | --- | --- | --- | --- | --- |
| **Sleep Time** | 1.08(1.01,1.16) | **0.03** | 1.08(1.01,1.16) | **0.03** | 1.01(0.94,1.09) | 0.83 |
| **Sleep Time** |  |  |  |  |  |  |
| 7-8 | ref | ref | ref | ref | ref | ref |
| <7 | 1.04(0.80,1.34) | 0.77 | 1.02(0.78,1.34) | 0.90 | 1.17(0.90,1.52) | 0.24 |
| >8 | 1.73(1.24,2.41) | **0.001** | 1.69(1.22,2.36) | **0.002** | 1.56(1.12,2.16) | **0.01** |

Model 1: No-adjust

Model 2: adjusted for age, sex, race, education, PIR

Model 3: adjusted for age, sex, race, education, PIR, smoking, drinking, physical activity, energy intake, BMI, WC, SBP, DBP, eGFR, uACR, total cholesterol, high‐density lipoprotein cholesterol, Diabetes, Hypertension, Cancer
