# Supplementary material for: Association of sleep time with all-cause and cardiovascular mortality among individuals with advanced cardiovascular-kidney-metabolic syndrome: A NHANES-based prospective cohort study
Source: Medicine (Baltimore). 2026 Oct 23;105(43):e51259. doi: 10.1097/MD.0000000000051259 (PMC13706387; doi:10.1097/MD.0000000000051259)
Supplement: Supplementary file 8 [file medi-105-e51259-s008.docx]

Supplementary Table S6.Excluding Patients with Cancer at Baseline

**ALL：**

|  | **Model 1**  **HR (95%CI)** | ***P* value** | **Model 2**  **HR (95%CI)** | ***P* value** | **Model 3**  **HR (95%CI)** | ***P* value** |
| --- | --- | --- | --- | --- | --- | --- |
| **Sleep Time** | 1.08(1.02,1.15) | 0.01 | 1.08(1.02,1.14) | 0.01 | 1.08(1.02,1.14) | 0.01 |
| **Sleep Time** |  |  |  |  |  |  |
| 7-8 | ref | ref | ref | ref | ref | ref |
| <7 | 0.91(0.74,1.12) | 0.35 | 0.89(0.73,1.09) | 0.25 | 0.89(0.72,1.09) | 0.26 |
| >8 | 1.35(1.07,1.71) | 0.01 | 1.28(1.02,1.60) | 0.04 | 1.26(1.01,1.59) | 0.04 |

Model 1: No-adjust

Model 2: adjusted for age, sex, race, education, PIR

Model 3: adjusted for age, sex, race, education, PIR, smoking, drinking, physical activity, energy intake, BMI, WC, SBP, DBP, eGFR, uACR, total cholesterol, high‐density lipoprotein cholesterol, Diabetes, Hypertension

**CVD：**

|  | **Model 1**  **HR (95%CI)** | ***P* value** | **Model 2**  **HR (95%CI)** | ***P* value** | **Model 3**  **HR (95%CI)** | ***P* value** |
| --- | --- | --- | --- | --- | --- | --- |
| **Sleep Time** | 1.05(0.97,1.15) | 0.23 | 1.06(0.97,1.15) | 0.21 | 0.98(0.89,1.07) | 0.62 |
| **Sleep Time** |  |  |  |  |  |  |
| 7-8 | ref | ref | ref | ref | ref | ref |
| <7 | 1.08(0.81,1.45) | 0.59 | 1.06(0.78,1.43) | 0.72 | 1.27(0.93,1.73) | 0.13 |
| >8 | 1.69(1.15,2.49) | 0.01 | 1.59(1.09,2.34) | 0.02 | 1.51(1.04,2.21) | 0.03 |

Model 1: No-adjust

Model 2: adjusted for age, sex, race, education, PIR

Model 3: adjusted for age, sex, race, education, PIR, smoking, drinking, physical activity, energy intake, BMI, WC, SBP, DBP, eGFR, uACR, total cholesterol, high‐density lipoprotein cholesterol, Diabetes, Hypertension
