# Supplementary material for: Association of sleep time with all-cause and cardiovascular mortality among individuals with advanced cardiovascular-kidney-metabolic syndrome: A NHANES-based prospective cohort study
Source: Medicine (Baltimore). 2026 Oct 23;105(43):e51259. doi: 10.1097/MD.0000000000051259 (PMC13706387; doi:10.1097/MD.0000000000051259)
Supplement: Supplementary file 9 [file medi-105-e51259-s009.docx]

Supplementary Table S4. Stratified analysis of the association between sleep time and mortality by NHANES survey cycles.

| **character** | **7 vs 7−8** | ***p*** | **7 vs 7−8** | ***p*** | ***p for trend*** | ***p for interaction*** |
| --- | --- | --- | --- | --- | --- | --- |
| All |  |  |  |  |  |  |
| Year |  |  |  |  |  | 0.567 |
| 2005-2014 | 0.907(0.794,1.036) | 0.152 | 1.251(1.032,1.515) | 0.022 | 0.31 |  |
| 2015-2018 | 0.84(0.633,1.114) | 0.226 | 1.306(1.062,1.771) | 0.007 | 0.239 |  |
| CVD |  |  |  |  |  |  |
| Year |  |  |  |  |  | 0.516 |
| 2005-2014 | 0.998(0.801,1.244) | 0.985 | 1.459(1.074,1.982) | 0.016 | 0.073 |  |
| 2015-2018 | 0.855(0.521,1.401) | 0.533 | 1.538(1.023,2.564) | 0.009 | 0.192 |  |
